# Xenobiotic-Metabolizing Enzymes in Trematodes

**DOI:** 10.3390/biomedicines10123039

**Published:** 2022-11-24

**Authors:** Viatcheslav Mordvinov, Maria Pakharukova

**Affiliations:** 1 Laboratory of Molecular Mechanisms of Pathological Processes, Institute of Cytology and Genetics SB RAS, 10 Akad. Lavrentiev Ave., Novosibirsk 630090, Russia; 2 Department of Natural Sciences, Novosibirsk State University, 2 Pirogov Str., Novosibirsk 630090, Russia

**Keywords:** liver fluke, Trematoda, ATP-binding cassette, cytochrome P450, glutathione S-transferase, anthelmintic, detoxification system

## Abstract

Trematode infections occur worldwide causing considerable deterioration of human health and placing a substantial financial burden on the livestock industry. The hundreds of millions of people afflicted with trematode infections rely entirely on only two drugs (praziquantel and triclabendazole) for treatment. An understanding of anthelmintic biotransformation pathways in parasites should clarify factors that can modulate therapeutic potency of anthelmintics currently in use and may lead to the discovery of synergistic compounds for combination treatments. Despite the pronounced epidemiological significance of trematodes, there is still no adequate understanding of the functionality of their metabolic systems, including xenobiotic-metabolizing enzymes. The review is focused on the structure and functional significance of the xenobiotic-metabolizing system in trematodes. Knowledge in this field can solve practical problems related to the search for new targets for antiparasitic therapy based on a focused action on certain elements of the parasite’s metabolic system. Knowledge of the functionality of this system is required to understand the adaptation of the biochemical processes of parasites residing in the host and mechanisms of drug resistance development, as well as to select a promising molecular target for the discovery and development of new anthelmintic drugs.

## 1. Introduction

The xenobiotic metabolism, which exists in all species, including parasitic ones, is responsible for homeostasis and the protection of the internal environment of the body when exposed to exogenous compounds, including various xenobiotics and anthelmintics. This review is aimed at determining the composition of the xenobiotic-metabolizing system in various trematodes and at assessing its structure, conservatism, and functionality.

Trematoda is a class within the phylum Platyhelminthes. There are no free-living trematodes, and all these species are internal parasites of mollusks and vertebrates. Most trematodes have a complex life cycle with two or three hosts. The life cycle is heterogonic with alternating sexual and asexual reproduction and with changes in generations and hosts. The definitive host, where the flukes sexually reproduce, is a vertebrate. The intermediate host, in which asexual reproduction occurs, is usually a snail. The class Trematoda or flukes includes plenty of species. The most epidemiologically important representatives include lung flukes *Paragonimus* spp.; liver flukes *Opisthorchis* spp., *Clonorchis sinensis*, and *Fasciola* spp.; and blood flukes *Schistosoma* spp. An estimated 200 million people may have a blood fluke schistosomal infection and 56.2 million people are infected with food-borne trematodes, which affect the lungs, liver, or intestines [1]. The hundreds of millions of people afflicted with schistosomiasis and other flatworm infections rely entirely on only two drugs (praziquantel and triclabendazole) for treatment. An understanding of anthelmintic biotransformation pathways in parasites should clarify the factors that can modulate the therapeutic potency of anthelmintics currently in use and may lead to the discovery of synergistic compounds for combination treatments.

At one point, it had been widely believed that all parasitic flatworms lack the cytochrome P450 system [2,3] and the entire first phase of xenobiotic metabolism. At the same time, information was published on the presence of oxidative metabolism of anthelmintic compounds in trematodes, along with evidence that drug metabolism is faster in resistant isolates [4,5,6,7]. These findings indicate the existence of a functionally active system for the biotransformation of exogenous and endogenous compounds in trematodes.

At present, large amounts of data have been accumulated during the implementation of projects for the sequencing of genomes and transcriptomes of epidemiologically significant trematodes. Nevertheless, there is still no adequate understanding of the functioning of many metabolic systems, including biotransformation and transport of exogenous and endogenous substrates. The knowledge of the functionality of this system is necessary to elucidate the adaptation of the biochemical processes of parasites to the residence in the host and the mechanisms of drug resistance development, as well as to select a promising molecular target for the discovery and development of new anthelmintic drugs.

This review of the structural and functional significance of the xenobiotic-metabolizing system in trematodes is also aimed at solving the fundamental issues of the organization of the “parasite–host” relationship. The knowledge in this field can solve practical problems related to the search for new target proteins for antiparasitic therapy based on a specific action on certain components of the parasite’s metabolic system. In addition, a parasite’s detoxification system is most likely responsible for the synthesis of parasite-specific genotoxic metabolites of cholesterol recently discovered in *Opisthorchis felineus* and other carcinogenic trematodes, *S. haematobium* and *Opisthorchis viverrini* [8,9].

## 2. Composition of the Xenobiotic-Metabolizing System in Trematodes

### 2.1. Phase I Enzymes

Phase I oxidation reactions are carried out by cytochrome P450 (CYP) enzymes, flavin-containing monooxygenases (FMOs), and epoxide hydrolases [10]. Other enzymes, such as aldo–keto reductases, aldehyde dehydrogenases, and alcohol dehydrogenases, are also involved in the first phase of xenobiotic metabolism.

CYPs in many species are the most extensively studied of all enzymes of xenobiotic metabolism because they are responsible for the metabolism of the vast majority of therapeutic drugs. CYPs are diverse in terms of the regulation of their gene activity and their catalytic activity. The number of genes in the genomes encoding CYP enzymes (CYPome) varies, ranging from a single isoform of an enzyme in some species of bacteria and fungi to dozens of isoforms in mammals and hundreds of CYP genes in plants. Currently, 102 functionally active CYP genes are known in mice and 57 in humans. CYPs contain a heme molecule as a cofactor. A heme is an oxygen-binding molecule that contains one iron atom at the center of the hydrocarbon structure. A heme performs the function of oxygen binding in the active site of the enzyme. CYP enzymes use an oxygen molecule, as well as an H^+^ ion, derived from NADPH to carry out the oxidation of substrates. H^+^ is supplied by the enzyme NADP-cytochrome P450 oxidoreductase, which works as a partner for cytochromes P450. Substrate metabolism by a CYP proceeds via the consumption of one molecule of oxygen and the formation of an oxidized substrate and a water molecule as a byproduct. The term P450 is derived from the spectrophotometric peak at the maximum absorption wavelength of the enzyme (450 nm) when it is in the reduced state as a complex with carbon monoxide.

Among the various reactions carried out by CYPs, the most well-known are the reactions of N-dealkylation, O-dealkylation, hydroxylation of aromatic hydrocarbons, N-oxidation, S-oxidation, and deamination. CYPs are involved in the metabolism of xenobiotics, as well as the synthesis of endogenous compounds, such as steroids and bile acids, which are byproducts of cholesterol breakdown. Mammalian xenobiotic-metabolizing CYPs have the ability to modify a large number of structurally different chemicals. This property is due to both the multiple forms of CYP and to the capacity of a single CYP to modify many structurally different compounds.

Flavin-containing monooxygenases (FMOs) are another phase I group of enzymes involved in xenobiotic metabolism. Similar to CYPs, FMOs are highly expressed and associated with the endoplasmic reticulum, the site in the cell where hydrophobic substrates interact and are metabolized. Epoxide hydrolases catalyze the hydrolysis of epoxides produced by CYP reactions. Epoxides are highly reactive and electrophilic; they can bind to cellular structures, proteins, RNA, and DNA, thereby leading to undesirable consequences for both the cell and organism. Epoxide hydrolases participate in the inactivation of potentially toxic derivatives.

Although CYPs are present in all kingdoms of living organisms, for a long time—due to unsuccessful attempts to identify them—it has been believed that parasitic flatworms lack both CYPs and the ability to oxidize xenobiotics [2,3]. Nevertheless, a CYP-like activity was later identified in some species of parasitic Platyhelminthes, the conversion of a number of drugs into their inactive metabolites has been reported [5,6,7], and evidence has been obtained about the role of such an activity in parasite drug resistance [2,4]. In particular, albendazole can be oxidized to albendazole sulfoxide in tissues of nematodes *Haemonchus contortus* [11], trematodes *Dicrocoelium dendriticum* and *Fascioloides vogae*, and the cestode *Moniezia expansa* [11]. *Fasciola hepatica* can metabolize triclabendazole to triclabendazole sulfoxide and triclabendazole sulfone, whereas the amount of metabolites is significantly increased in resistant strains [4]. Moreover, if resistant strains of *Fasciola* are incubated with ketoconazole (an inhibitor of CYPs), then these helminths become sensitive to the anthelmintics. All these data indirectly indicated the presence of a CYP system in parasitic flatworms.

**Figure 1:**
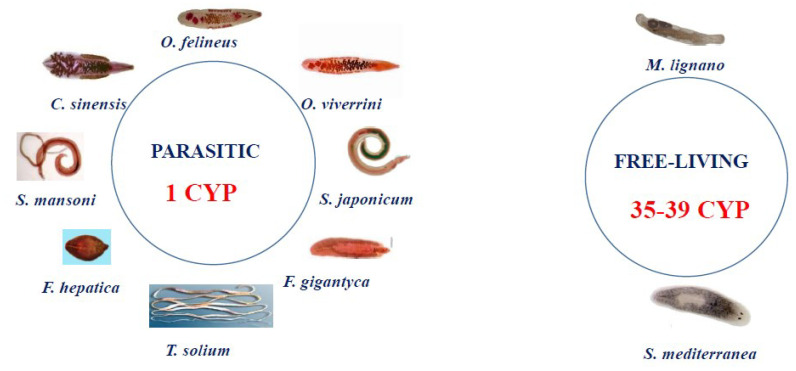
In silico data: composition of the CYPome in Platyhelminthes. Only one CYP gene has been found in all known parasitic flatworms. The following sequences were analyzed: *S. mansoni* (Sm) CYP GenBank ID: KT072747.1; *O. viverrini* CYP GenBank ID: XM_009163872.1; *S. haematobium* CYP GenBank ID: XM_051214700.1; *Schistosoma japonicum* CYP GenBank ID: AY815629.1; *F. hepatica* CYP GenBank ID: GEVX01002683.1; *Taenia solium* CYP GenBank ID: JZ063849.1; *F. gigantica* CYP GenBank ID: TPP59328.1; and *C. sinensis* CYP GeBank ID: KAG5450480.1. *O. felineus* CYP homolog sequences from free-living Platyhelminthes *S. mediterranea* and *M. lignano* were retrieved from the Genome Database (https://planosphere.stowers.org/smedgd (accessed on 21 November 2022)) and the Genbank NCBI (GeBank ID: PRJNA284736), respectively.

Analysis of the available data on the genomes and transcriptomes of parasitic and nonparasitic flatworms revealed that the CYPome drastically differs between these two groups. Although the free-living species have dozens of weakly homologous diverged CYP genes (for example, 39 CYPs in *Schmidtea mediterranea*), parasitic species (Schistosomatidae, Opisthorchiidae, Taeniidae, and Fasciolidae) have only one cytochrome P450 [12,13,14] (Figure 1).

Analysis of the coding region of the P450 sequence revealed the presence of a functional Pfam00067 CYP domain (E-value = 3.75 × 10^−29^) characteristic of eukaryotic microsomal type II CYPs [12]. This type of CYP is widespread among many organisms and is directly involved in the biotransformation of exogenous compounds: xenobiotics and drugs. This type of CYP is located in the membrane of the endoplasmic reticulum and functions in tandem with NADPH CYP reductase, which is necessary for electron transfer to cytochrome P450 from NADPH. The gene-encoding NADPH CYP reductase has been found in genomes of parasitic Platyhelminthes [2]. In the *O. felineus* (Of) CYP structure, a transmembrane domain was also found in the N-terminal region [12].

**Figure 2:**
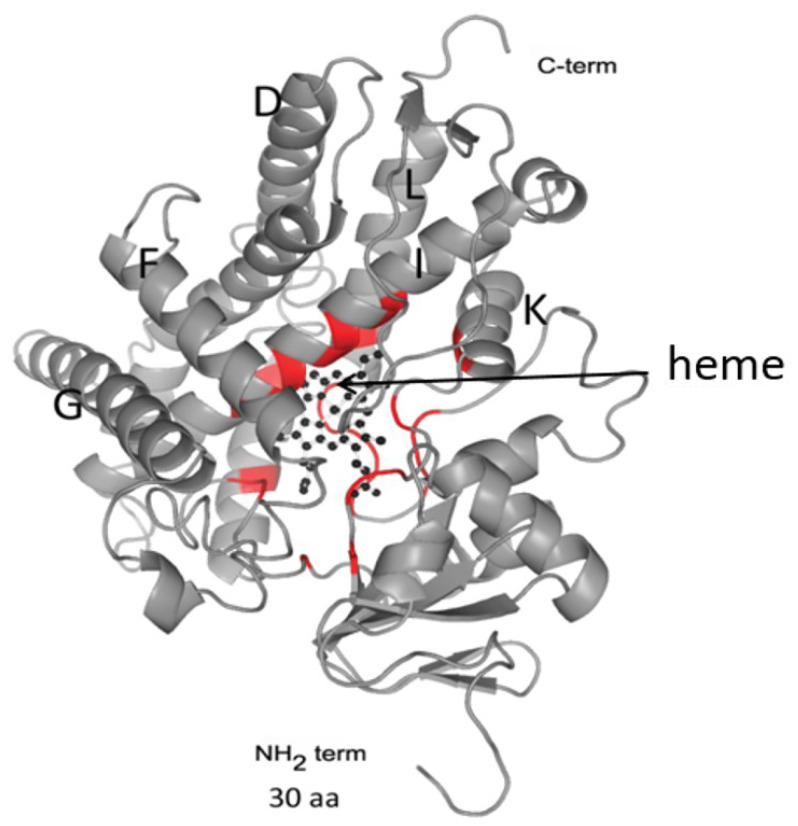
A 3D structure modeling of OfCYP (Phyre2). The arrow indicates the position of the heme within the globular region of the protein. Regions of the protein taking part in heme positioning are highlighted in red.

OfCYP shares high homology (91%) with CYP of *C. sinensis* and *O. viverrini*, lower homology with that of Schistosomatidae (29–37%), and low homology with CYPs of free-living flatworm species (23–24%) [12,13]. Similar results indicating low homology (20–24%) with Eumetazoa CYPs were also reported for *S. mansoni* CYP [15]. Despite the low homology of the primary sequences, CYPs have a conserved folding of small regions, which implements the function of monooxygenase catalysis. Conserved regions are located predominantly in the C-terminal region of the protein and form a four-helical globule (D, E, I, L), J and K α-helices, two β-fold regions, and a loop called a “meander” (Figure 2). This region contains a heme-binding loop, with the characteristic P450 consensus motif Phe-X-X-Gly-X-Arg-X-Cys-X-Gly (Phe400-Ser-Leu-Gly-Ala-Arg-Ser-Cys-Pro-Gly409 in OfCYP) [12].

In addition, it has been demonstrated that *O. felineus* detoxification phase I is also represented by genes encoding aldo–keto reductases, aldehyde dehydrogenases, and alcohol dehydrogenases [14]. The expression of all aldehyde dehydrogenases is higher in the adult stage than in metacercariae, while the expression of aldo–keto reductases is almost at the same level in both stages [14].

Interestingly, liver flukes lack the group of enzymes having a monooxygenase activity (analogous to CYPs), namely, flavin monooxygenases (Pfam00743) and epoxide hydrolases (PF00702). Moreover, we failed to find any flavin monooxygenase sequences when searching the nucleotide sequence database of the related parasitic flatworm species, including opisthorchiids, schistosomes, and fasciolids [14]. In contrast, flavin monooxygenase genes are present in nematode genomes [16].

### 2.2. Phase II

Phase II enzymes are responsible for the elimination of xenobiotics and drugs from the body and for the inactivation of electrophilic and potentially toxic metabolites produced by phase I enzymes. Phase II reaction products are metabolites with improved water solubility and increased molecular weight. The conjugation reactions that these enzymes perform occur when a substrate has an oxygen-containing group (hydroxyl or epoxy groups), an amino group, or sulfur atoms that serve as an acceptor for a hydrophilic moiety of such molecules as glutathione, glucuronic acid, or sulfate or an acetyl group that is covalently conjugated to an acceptor site on the xenobiotic molecule. Phase I oxidation by enzymes either adds or modifies a functional group, allowing the resulting reaction products to serve as substrates for phase II conjugating enzymes. As a consequence, the metabolite, which is at this point more soluble in water and has higher molecular weight, is excreted in urine or bile. Enzymes associated in phase II of xenobiotic metabolism include several superfamilies of conjugation enzymes. The most important of these are glutathione S-transferase (GST), sulfotransferase (SULT), UDF-glucuronosyl transferase (UGT), arylamine N-acetyltransferases, glycine-N-acyltransferase (GLYAT), methyl transferase, and glutathione peroxidase.

GSTs catalyze glutathionylation by adding glutathione (GSH) to an electrophilic center of their substrates [17]. They can also reduce lipid peroxidation products formed by a free-radical attack on water-soluble compounds [18]. GSTs are categorized into four major classes based on substrate specificity: cytosolic GSTs, kappa-class GSTs (mitochondrial), membrane-associated proteins of eicosanoid, and glutathione metabolism [19]. The cytosolic GSTs are more abundant and can be further subdivided into several classes, such as mu, alpha, pi, theta, sigma, zeta, omega, nu, lambda, phi, tau, delta, epsilon, iota, chi, and rho [17,20,21]. Structurally, most of the GSTs are dimeric and can be either a homodimer or heterodimer.

The general reaction consists of conjugating the reduced GSH to molecules with an electrophilic center (1), including alkyl and aryl halides, carboxylates, sulphate and phosphate esters, epoxides, organic nitrates, lactones, quinones thiocyanates, and hydroperoxides [3,19].

GSH + RX → GSR + HX(1)

Genes encoding phase II enzymes are widespread in the genomes of flat and round parasitic worms [22,23,24]. The main cytosolic GST classes identified in helminth parasites are mu, pi, and sigma, along with some alpha and omega class GSTs [22,23,24,25].

**Figure 3:**
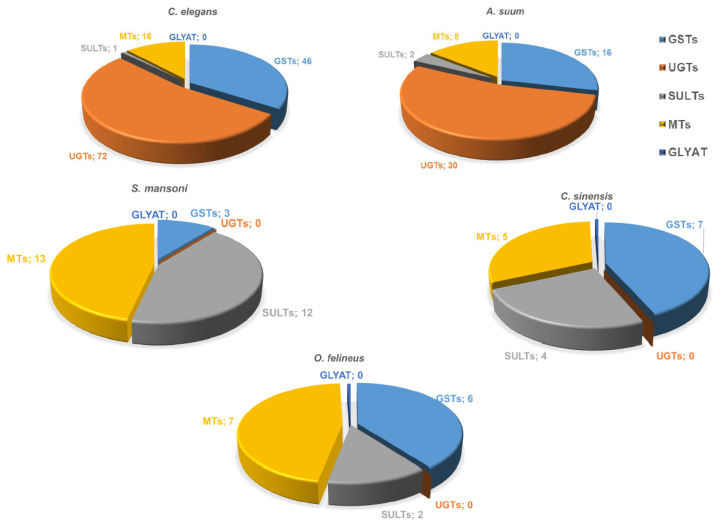
The spectrum of annotated nematode and trematode genes containing conserved domains of “conjugation phase II” proteins. GST, glutathione S transferase; SULT, sulfotransferase; UGT, UDP-glucuronosyl transferase; GLYAT, glycine-N-acyltransferase; and MT, methyltransferase. Numbers represent the number of genes identified in the genomes.

Sixteen GST genes have been identified in *A. suum* nematodes, and 46 genes in *C. elegans*, while 5–7 GST genes have been identified in opisthorchiids, and three genes in *S. mansoni* (Figure 3). The *O. felineus* genome contains six GST genes, which have the highest expression among all detoxification genes. The 28 kDa GST sigma is especially highly expressed; its mRNA abundance in the adult worm is by two–three orders of magnitude higher as compared with the other detoxification genes [14]. UGTs (common phase II xenobiotic metabolism enzymes in vertebrates enhance hydrophilicity and availability of substrates to efflux transporters. The UGT superfamily comprises over 20 isozymes.

A comparative study on the composition of conjugation phase II enzymes in various parasitic worms as well as free-living worms has been aimed at identifying sequences corresponding to conjugation phase II proteins in the available data on genomes and transcriptomes of Opisthorchiidae and *S. mansoni* flatworms as well as in roundworms: parasitic species *A. suum* and free-living *C. elegans* [14]. It turned out that UDP-glucuronosyl transferases, which are the main enzymes of phase II, both in parasitic and free-living roundworms, are completely absent in flatworms. Currently, 34 UGT genes are known to be present in the nematode *H. contortus* genome [26], and 72 UGT genes in the *C. elegans* genome [27].

In addition, we did not find any arylamine N-acetyltransferases (PF00797) [14], which are also common phase II xenobiotic metabolism enzymes in vertebrates. Thus, detoxification phase II of parasitic flatworms has essential specific structural and functional features distinguishing it from the corresponding systems in other organisms, including the host system performing metabolism of exogenous substrates (Figure 3). As for methyltransferases, genes encoding these enzymes are present in both trematode and nematode genomes; e.g., 8 genes have been identified in *A. suum* and 16 genes in *C. elegans*, while 5–7 genes have been identified in opisthorchiids and 13 in *S. mansoni* (Figure 3). The genes encoding sulfotransferases are present in both trematode (5–13 genes) and nematode genomes (1–2 genes) (Figure 3). It is noteworthy that no glycine-N-acyltransferase (GLYAT) genes have been found in either flatworms or roundworms.

### 2.3. Identification of Phase III Genes

The removal of xenobiotics and drugs from the cell is implemented by the cell’s excretory system. The main xenobiotic efflux proteins are ATP-binding proteins (ABC, i.e., ATP-binding cassette) and OATP2 proteins (organic anion transporting polypeptide 2) [28]. ATP-binding proteins have been found in all animal and plant species from prokaryotes to eukaryotes [29]. ABC proteins have a conserved structure, several transmembrane domains, and broad substrate specificity. In mammals, these proteins carry out an ATP-dependent transport of toxic compounds and drugs from the cell to the extracellular space across the membrane.

Although there are differences in their function and types of substrates, they share high structural homology. Human P-glycoprotein (ABCB1), which is encoded by the *MDR1* gene, is the best-known and best-studied ABC transporter among human ABC proteins. ABCB1 was discovered in 1976 [30] because of its involvement in the multidrug resistance of cancer cells to chemotherapy, which is how it got its name (multidrug resistance gene, MDR). The protein is made up of two parts that share high similarity. Each homologous region contains six hydrophobic transmembrane domains and a hydrophilic intracellular region encoding an adenosine triphosphate (ATP)-binding site (nucleotide-binding domain, NBD).

**Figure 4:**
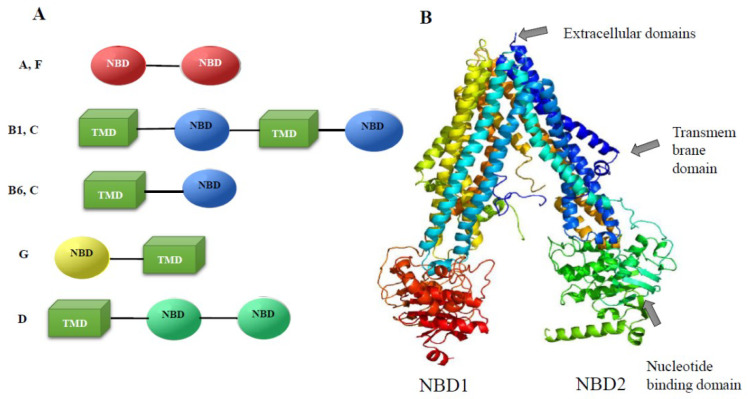
Structure of conserved domains of *O. felineus* ABC proteins. (**A**) The structure of ABC proteins is presented. TMD, transmembrane domain; NBD, nucleotide-binding domain. (**B**) The Pgp4 3D model (Phyre2 multi-matrix simulation). The (rainbow-colored) model was built using Phyre 2 multi-matrix modeling. The matrices selected by the Phyre algorithm for modeling were P-gp from *C. elegans* (4F4CA) and P-gp1 from *Mus musculus* (3G5U). The structure of a region of 1202 amino acid residues (95% of the P4 sequence) was modeled at a 100.0% match, although a region of 45 amino acid residues (aa 612–656) was modeled ab initio at a low match.

The genes of this family are common in genomes of parasitic flat and round worms. In particular, 20 genes in the genome have been detected in the parasitic flatworm *S. mansoni* [31], and 23 genes of transporters of the ABC family were identified in the *O. felineus* genome [32]. In particular, the genome contains four genes of the A-subfamily, eight genes of the B subfamily, five ABCC genes (multidrug resistance associated protein), one ABCD, two ABCF, and three ABCG genes (Figure 4A).

The organization of conserved regions in the coding regions of the ABC transporters differs among the subfamilies of these proteins (Figure 4A). In particular, complete transporters composed of two successively alternating transmembrane and nucleotide-binding domains are present only in the ABCC subfamily (five proteins) and ABCB subfamily (six proteins). This domain organization is conserved and is present in mammals, plants, and roundworms. The structure of the predicted semitransporters of the ABCG and ABCB6 subfamilies, which include only one transmembrane domain and one NBD, also matches the structure of homologs in mammals. The structure of transporters of the ABCA subfamily (four proteins), which do not contain transmembrane domains, is noteworthy, while in mammals these are complete transporters. Their function is the transport of lipids and signaling molecules. Obviously, in trematodes, due to the absence of transmembrane domains in ABCA, the transport of lipid compounds is performed by transporters of other subfamilies.

All four *MDR* genes (ABCB1) of *O. felineus* have homologs in other species of liver flukes. The degree of homology with those of *C. sinensis* and *O. viverrini* is 94–97% [32]. A 3D model of the *O. felineus* Pgp4 (MDR, ABCB1) protein (Figure 4B) built using Phyre2 multi-array modeling indicates conserved structure of this protein. The matrices selected by the Phyre algorithm for modeling were P-gp from *C. elegans* (4F4CA) and P-gp1 from *Mus musculus* (3G5U). The structure of a region of 1202 amino acid residues (95% of the P4 sequence) was modeled at a 100.0% match [32].

## 3. Functionality of Trematoda Xenobiotic-Metabolizing Enzymes: A Potential Link to Drug Resistance

### 3.1. Functionality of Phase I Enzymes

To date, significant data have been accumulated indicating that cytochromes P450 play an important role in the physiological processes and survival of many organisms. In particular, in protozoa, *Leishmania donovani* CYP has been found to be essential for survival, invasiveness, and ergosterol biosynthesis [33]. It has been shown that the removal of one allele from the *Leishmania* genome leads to altered membrane potential, growth anomalies, reduced invasiveness, and increased sensitivity to drugs [34]. If two alleles of the CYP450 gene are deleted, then the parasite is unable to survive [34]. According to data on *C. elegans* roundworms, one of the P450 cytochromes participates in the synthesis of the steroid hormone necessary for development into an adult [35], while the other P450 is involved in homeostasis and storage of fatty acids [36], i.e., it also performs functions important for homeostasis and survival. In fungi, CYP is known to be involved in the synthesis of the spore cell wall, the metabolism of membrane sterols, and the production of metabolites with antibacterial activity [10]. To date, the functionality of trematode CYP has been shown indirectly, using in silico mathematical modeling and in situ imaging of oxidative metabolism [13,15,37]. Nevertheless, it is clear from the obtained data that CYP is important for viability [13,15] and for benzimidazole biotransformation [6,7].

During the isolation of the microsomal fraction of an *O. felineus* adult worm, CYP was extracted in the inactive P420 state [15]. This form is typical of the inactive low-spin state of CYP420 [38,39]. Indeed, measurement of activities related to O-hydroxylation of substrates (ethoxy-, methoxy-, benzoxy-, and pentoxyresorufins) in the microsomal fraction did not reveal the enzymatic activity [13]. In contrast, there is evidence that microsomes from *S. mansoni* and *S. haematobium* retain their enzymatic activity when isolated [5]. Nevertheless, no other literature source mentions this. Many cases are known when CYPs are released in the low-spin state CYP420 [38,39,40,41]. The transition from a high-spin CYP to a low-spin state is caused by a change in the position of the heme iron atom in the cytochrome active site [38]. The protonation of cysteine to thiol can cause the P450–P420 transition [40,41].

The regulation of *Ofcyp* mRNA expression was studied using exogenous substrates in adult worms and excysted metacercariae (dimethyl sulfoxide, tetrachlorodibenzodioxin, phenobarbital, dexamethasone, ethanol, and ketoconazole). To the six types of inducers, we also added biological inducers: endogenous fluids of the hamster blood serum and hamster bile and hemoglobin. These substances did not affect the level of *Ofcyp* mRNA upon treatment for 4 and 24 h [13].

Given that it was not possible to isolate OfCYP in the active state, the ability of the liver fluke *O. felineus* to metabolize substrates of CYP in situ was tested when substrates were added to the medium [13]. By microscopy and HPLC, results were obtained indicating that *O. felineus* is able to metabolize chlorzoxazone (a substrate specific for mammalian CYP2E1 monooxygenases), as well as fluorogenic alkoxyresorufins (pentoxyresorufin and benzoxyresorufin but not methoxyresorufin). Large aggregates of the product (resorufin) ~5 μm in size were found in the excretory system of the adult liver fluke [13]. Interestingly, there was no fluorescent staining in the gut of the parasite. Ketoconazole inhibited the accumulation of fluorescent products, thus indirectly supporting the idea that the formed fluorescent substance in the tissues is a product of CYP. Structure-based screening and molecular dynamics simulation of OfCYP confirmed that some azole substances have significant potential to inhibit OfCYP450 [37].

The suppression of CYP mRNA expression in adult *O. felineus* worms by RNA interference using double-stranded RNA decreases the mRNA expression of the CYP gene by 62–70% within 8 days [13]. At the same time, worms with suppressed expression of CYP demonstrated a high mortality rate (60% at 8 days after the knockdown). Thus, the *O. felineus* CYP participates in the metabolism of exogenous substrates, is important for survival of adult individuals, and represents a promising target for anthelminthic therapy [13,42].

Similar results were obtained in a study on *S. mansoni* CYP [15]. A *S. mansoni* (Sm) CYP knockdown using RNA interference in juvenile worms resulted in worm death. The treatment of juvenile or adult worms with azole CYP inhibitors revealed anthelmintic effects at low micromolar concentrations. Furthermore, a combination treatment of worms with an anti-SmCYP double-strand RNA probe and miconazole manifested additive killing effects on the parasites. Moreover, miconazole inhibited the embryogenesis in developing *S. mansoni* eggs. Therefore, these data revealed that SmCYP is the target of miconazole and is essential for parasite survival and egg development [15].

In roundworms, more data have been accumulated on the functionality of P450 cytochromes and their role in drug metabolism. For instance, the treatment of larvae of both the nematodes *Cooperia oncophora* and *Ostertagia ostertagi* with piperonyl butoxide, which is an inhibitor of CYP, made the larvae more sensitive to anthelmintics, in particular macrocyclic lactones. These findings suggest that cytochromes P450 play a part in the metabolism of thiabendazole and macrocyclic lactones, such as ivermectin [43].

Interestingly, the liver fluke *D. dendriticum* metabolizes albendazole to sulfoxide derivatives; in addition, it has been shown that other drugs can also undergo biotransformation in its tissues; in particular, mebendazole and flubendazole are either reduced or reduced and methylated [2]. It has been demonstrated that triclabendazole is converted into sulfone and sulfoxide derivatives in *F. hepatica*. Moreover, in resistant strains, these biotransformed products have been detected at higher concentrations [6,7]. The metabolism of flubendazole and mebendazole was also demonstrated in the *Moniezia expansa* cestodes, and oxidation to albendazole sulfone and sulfoxide was noted for albendazole [44]. The biotransformation of various benzimidazoles in nematodes has been documented, in particular, in *A. suum* and *H. contortus* [7].

### 3.2. Functionality of Phase II Enzymes

In humans, cytochrome P450s and UDP-glucuronosyl transferases together are responsible for clearing more than 90% of all prescription drugs [10,28]. In invertebrates, phase II enzymes of metabolism are also responsible for drug metabolism. In particular, glutathione S-transferases play an important role in the detoxification of flumethrin and chlorpyrifos in the tick *Haemaphysalis longicornis* [45]. Double stranded RNA induced silencing of the *GST* gene increases susceptibility of this species to these anti-tick agents [45].

In helminths, GSTs are involved in the detoxification of lipid hydroperoxides and carbonyl compounds produced in large quantities during oxidative stress. Genes of these enzymes are present in the genomes of nematodes and trematodes, and the activity of some recombinant proteins has been studied. Moreover, there is evidence that GSTs mostly have functions associated with survival under conditions of moisture deficiency (for *H. contortus*), as well as resistance to thermal and oxidative stress (for *C. elegans*) [11].

Many GSTs from helminths, such as *Schistosoma* and *Clonorchis* species, have been characterized [22], and crystal structures are available for several of them [23,24]. The ability to metabolize drugs in vitro has been shown for GST in helminths. Research on the recombinant mu-class GST1 from *Fasciola gigantica* [46] revealed that this enzyme forms a homodimer and possesses broad substrate specificity. In particular, the activity of the conjugation of glutathione under the action of this enzyme was documented toward 1-chloro-2,4-dinitrobenzene, 4-nitroquinoline-1-oxide, and trans-4-phenyl-3-buten-2-one.

The antioxidant system seems to be important for the protection of *S. mansoni* sporocysts from exogenous oxidative stress [47]. In particular, a knockdown of GST26, −28, Prx1/2, and GPx by RNA interference for 7 days was performed. This intervention resulted in significantly increased susceptibility to oxidative stress caused by oxygen peroxide. Consequently, these data suggest that antioxidant defense proteins are important for protection against exogenous oxidative stress. *C. sinensis* GST omega proteins may be crucial for the protection of the reproductive system during the maturation of *C. sinensis* worms and in response to oxidative conditions, thereby contributing to the maintenance of parasite fecundity [22]. A GST knockdown via RNA interference in *S. japonicum* worms resulted in a silencing rate higher than 80% [48]. The egg reduction rate (18%) and abnormal egg ratio (28%) were significantly higher in the GST-deficient group than in the negative control group. These results support the notion that parasitic GST plays an important part in the fecundity of trematodes, specifically in egg formation [48].

UGTs play a major role in the drug resistance of roundworms [11,49]. Glucose conjugation is one of the main pathways for the biotransformation of benzimidazoles, such as albendazole and flubendazole, in the roundworms *H. contortus* and *C. elegans* [11]. In particular, glucose conjugation is a major pathway of xenobiotic metabolism in *C. elegans*, and this enzyme may be a target for the enhancement of anthelmintic potency [50]. In particular, the biotransformation of albendazole by *C. elegans* reduced drug potency in that study. Glucose metabolite production by *C. elegans* was low in the presence of the pharmacological inhibitor chrysin, suggesting that UDP-glucuronosyl/glucosyl transferase (UGT) enzymes may catalyze benzimidazole glucosidation. The resistant strains of *H. contortus* produced much more of the glucose–albendazole and glucose–flubendazole conjugates. Furthermore, the transcriptomic response was assessed after the treatment of resistant strains of *C. elegans* with benzimidazole anthelmintic agents (albendazole, mebendazole, thiabendazole, and oxfendazole). It is noteworthy that significant upregulation of classic xenobiotic metabolism genes, including UGT enzymes, was revealed [50]. Moreover, the treatment of worms with each of the five anthelmintics resulted in upregulation of a common subset of 41 genes. This group included 10 genes of cytochrome P450s, 8 genes of UDP-glycosyltransferases, and two genes of GSTs, as well as genes of oxidoreductases, dehydrogenases, and reductases. Consequently, the results of this transcriptomic study suggest that glucose conjugation is a major pathway of biotransformation in nematodes. Moreover, the glucose conjugation pathway may be a target for the enhancement of anthelmintic potency. In another work, by means of the UGT inhibitor 5-nitrouracil, it was possible to increase susceptibilty of resistant isolates of the sheep nematode *H. contortus*, to the anthelmintic naphthalophos [51]. Thus, these data indicate that enzymes of xenobiotic metabolism are promising as novel targets for increasing the anthelmintic activity in nematodes.

The role of UDP-glucuronosyltransferases in the detoxification of naphthalophos by *H. contortus* larvae was shown by the phenobarbital induction of xenobiotic detoxification enzymes in *H. contortus* [51]. The phenobarbital-induced drug tolerance was reversed by cotreatment with UDPGT inhibitors 5-nitrouracil, 4,6-dihydroxy-5-nitropyrimidine, probenecid, or sulfinpyrazone. The UDPGT inhibitors, 5-nitrouracil, 4,6-dihydroxy-5-nitropyrimidine, and probenecid, also exerted synergistic effects in non-phenobarbital-treated worms (synergism ratio up to 3.2-fold). These findings lend support to a chemotherapeutic approach based on inhibitors of UDPGT enzymes as synergists to increase the activity of naphthalophos against parasitic worms and to combat detoxification-mediated drug resistance if it arises in the field [51].

A striking example of the involvement of xenobiotic-metabolizing enzymes in the resistance of trematodes to anthelmintics is the naturally selected drug resistance of schistosomes to oxamniquine. Oxamniquine resistance in schistosomes has a recessive basis and results in up to 500-fold reduction in drug sensitivity [52,53,54]. Quantitative trait locus (QTL) analysis was applied to discover a chromosome locus responsible for drug resistance in *S. mansoni*. Thus, the cross hybridization of a sensitive and a resistant parasite and subsequent genetic analysis revealed a single QTL on chromosome 6. According to further analysis, a sulfotransferase (Genbank ID: Smp_089320) was identified as the causative gene. Moreover, RNAi knockdown confirmed the involvement of sulfotransferase Smp_089320 in oxamniquine-resistance in parasites using double-stranded RNA probe targeting of the identified gene. As a result, sensitive parasites became resistant [54]. Crystal structure analysis of the sulfotransferase from resistant worms revealed mutation-induced perturbations of the enzyme structure that abrogate oxamniquine binding and/or sulfonation [54].

The involvement of the xenobiotic-metabolizing enzyme system in the metabolism of xenobiotics has been shown in the nematode *Bursaphelenchus xylophilus*. For instance, out of 51 genes of the xenobiotic-metabolizing enzyme system in the genome, including flavin monooxygenase (FMO), cytochrome P450 (CYP), short-chain dehydrogenase (SDR), alcohol dehydrogenase (ADH), aldehyde dehydrogenase (ALDH), UDP-glucuronosyltransferase (UGT), and GST [55], some are differentially expressed in response to treatment with nematicides. In particular, β-pinene-activated detoxification enzymes (cytochrome P450, UDP-glucuronosyltransferases, and short-chain dehydrogenases), ion channel/transporter activity (ATP-binding cassette families), and nuclear-receptor-related genes [16].

Unfortunately, in general, there is very little information about these enzymes in other flatworms. The sheep tapeworm *Moniezia expansa* was used to assess the activity of the main drug-metabolizing enzymes and evaluate the metabolism of selected anthelmintics (ABZ, MBZ, and FLU) in *M. expansa* [44]. The activities of biotransformation enzymes were determined in subcellular fractions, but no conjugated metabolites of anthelmintics were identified either ex vivo or in vitro.

Thus, the metabolism of antiparasitic agents is fundamentally different between two types of worms (Platyhelminthes and Nematoda phyla), which must be taken into account during research into the mechanisms of resistance to anthelmintic therapy.

It is important to note that many enzymes of the conjugation phase in trematodes, in particular liver flukes, are components of the excretory–secretory product; e.g., several GSTs are secreted in large quantities into the incubation medium and are detectable in epithelial cells of mammalian bile ducts [56,57]. Given the high level of expression, the *Ofgsts* gene in adult *O. felineus* worms is among the 10 most abundant genes (GenBank ID: GBJA01007477.1) [14], and it is likely that conjugation phase enzymes are involved in the molecular mechanisms of the host–parasite relationship. Thus, the role of conjugation phase proteins may turn out to be more important than mere participation in the neutralization of xenobiotics and could be important for characterizing the system of parasite–host relationships in chronic trematodiases.

### 3.3. Functionality of Phase III Proteins

To date, a relation between drug resistance in nematodes and the expression of P-glycoproteins (MDR proteins) in these worms has been revealed, in particular, in *Cooperia oncophora, Brugia malayi*, and *C. elegans* [58]. In addition, a knockout of individual P-glycoproteins in free-living roundworms *C. elegans* leads to a significant increase in sensitivity to anthelmintic drugs [59]. Moreover, if *C. elegans* is treated with successively increasing concentrations of ivermectin, then isolates with increased expression of these genes and drug resistance can be obtained [60]. Resistance to ivermectin is not implemented by one gene, this is a multigene trait; however, the use of ABC transporter inhibitors, such as verapamil, raises drug sensitivity in resistant strains of *C. elegans*, *H. contortus*, *C. oncophora*, *T. circumcincta*, and *O. ostertagi* [43,51]. In trematodes, the functionality of ABC efflux pumps can be deduced only from the data of in silico modeling [61,62]. Docking simulation showed that CsMRP4 is able to transport bile acids [61,62]. Nevertheless, a direct relation between the activity of ABC transporter proteins and susceptibility to praziquantel has been demonstrated [31,63,64].

Although there are much less data on trematodes, it is believed that activities of this particular family of proteins are associated with the excretory system of trematodes [65]. A specific feature of this system in parasites is also its key role in the system of host–parasite interactions, which determines a variety of pleiotropic pathogenic effects on the host organism. To visualize the activity of P-glycoproteins, we chose resorufin, a low-molecular-weight fluorescent substance, which, as previously shown, is a substrate for P-glycoproteins [65]. Thus, by visualizing the workings of the excretory system of flatworms with the help of resorufin, one can see the functioning and activity of exporter proteins and test various inhibitors. It is reported that resorufin penetrates evenly through the tegument and accumulates in excretory tubules; then, resorufin is concentrated in the excretory bladder and excreted through the excretory pore. Praziquantel has been shown to act as a substrate for ABC transporters. Because of the shared structure of transporters with mammalian protein homologs, the use of such inhibitors as verapamil and tariquidar is believed to be justified. The application of the inhibitors tariquidar and verapamil simultaneously with resorufin to flatworms in vitro revealed that the inhibitors prevent the accumulation of resorufin in the excretory tubules but do not prevent the dye from entering the worm [32,65]. Thus, resorufin enters the worm tissues most likely by diffusion through the tegument, and P-glycoproteins are implicated in the elimination of this drug through the excretory system. In addition, these results demonstrate that P-glycoproteins are functionally and structurally very similar to mammalian proteins, and these inhibitors or their derivatives can probably be helpful for increasing the concentration of the drugs inside the worm and enhancing the action of anthelmintic drugs.

Moreover, in *S. mansoni*, it was shown that SMDR2 is a P-glycoprotein homolog, and its expression goes up in response to drugs [63]. In praziquantel-resistant isolates of *S. mansoni* schistosomes, the expression of the *SMDR2* gene encoding a protein homologous to P-glycoprotein is significantly increased. The inhibition or knockdown of ABC transporters enhances the susceptibility of adult and juvenile schistosomes to praziquantel [31,63]. Moreover, a comparison of efflux pumps’ activities in praziquantel-susceptible and resistant *S. mansoni* strains revealed that low doses of verapamil successfully reverse drug resistance [64]. Thus, a direct relation between the activity of ABC transporter proteins and sensitivity to antiparasitic drugs in schistosomes has been demonstrated.

Triclabendazole-resistant isolates of *F. hepatica* are widespread throughout the world. In resistant isolates, elevated expression and activity of ABC transporters, as well as the presence of mutations in the coding region have been observed [66,67]. Nonetheless, it was later shown that various SNPs in the *F. hepatica* P-glycoprotein gene from diverse Latin American and Australian populations of fasciolids are not associated with triclabendazole resistance [66,67].

## 4. Xenobiotic-Metabolizing Enzymes as Targets for Anthelmintic Therapy

### 4.1. Phase I Enzymes

Some proteins of the detoxification system are important pharmacological targets because they are critical for their own metabolism and the survival of pathogens. In particular, this topic primarily concerns cytochromes P450. More than 20 years ago, based on a fungal cytochrome P450 inhibitor, fungicide drugs were developed that are effective as fungicidal agents and are currently used in medicine [10]. These are azole compounds, such as ketoconazole, fluconazole, and other imidazole derivatives. It is known that *Trypanosoma cruzi* CYP51 is an important pharmacological target, and clinical trials of compounds based on *T. cruzi* CYP inhibitors are currently underway [68]. Previously, these compounds have been successfully tested in the treatment of trypanosomiasis in model animals. Lately, the search for new drugs against trematodiases has been actively conducted [1], both by the screening of libraries of chemical compounds and by researching parasitic proteins as new molecular targets for the development of targeted drugs.

From this point of view, cytochrome P450 can be a promising target for the development of drugs against trematodiases. From the evidence of a decrease in the survival rate of liver flukes subjected to a CYP gene knockdown [13,15], it can be concluded that this gene is important for parasite viability. Because all parasitic flatworms, including liver flukes (Opisthorchiidae and Fasciolidae), blood flukes (Schistosomatidae), and cestodes (Taeniidae) have only one CYP gene [12], it is possible to suppress CYP monooxygenase activity by means of selective inhibitors.

The testing of the anthelmintic activity of universal azole CYP inhibitors—ketoconazole, miconazole, triadimenol, clotrimazole, 4-phenyl imidazole, and others—on *O. felineus* and *S. mansoni* trematodes indicates that some of these compounds actually have promising anthelmintic activity against both juvenile and adult parasites. Such an activity has been documented in vitro using micromolar drug concentrations in both motility and survival tests [15,42]. The most effective drugs in this regard are miconazole and clotrimazole [15,32]. At the same time, miconazole has proven to be as effective as praziquantel against *O. felineus* and *S. mansoni* and causes 100% mortality of worms at a dose of 5–10 µM [15,42]. In addition, other inhibitors of heme-containing enzymes with non-azole structures were tested: disulfiram, metyrapone, benzyl isothiocyanate, and ticlopidine [42]. The evaluation of these substances by standard motility tests on excysted metacercariae showed that some of the compounds are quite promising.

Moreover, a synergistic interaction was demonstrated for a praziquantel–clotrimazole (CI = 0.68) in vitro combination and for a praziquantel–miconazole (CI = 0.68) in vitro combination against adult helminths [69]. Praziquantel and miconazole (CI = 0.30) had a strongly synergistic effect against newly excysted *O. felineus* metacercariae [69]. Unfortunately, the synergistic effects of the praziquantel–clotrimazole combination and praziquantel–miconazole combination observed in vitro were not confirmed in vivo.

The treatment of hamsters infected with juvenile worms (1 day postinfection) resulted in a worm burden reduction of 37.5%, with 100 mg/kg clotrimazole killing 31.25% of the worms [69]. At 5–6 weeks postinfection, which corresponded to the infection with adult worms, the treatment of hamsters with miconazole yielded a worm death rate of 23.8% and 21.4%, respectively. The administration of praziquantel together with clotrimazole or with miconazole killed worms slightly more frequently than praziquantel alone did (59.5% and 54.7% versus 50% for praziquantel).

### 4.2. Phase II Enzymes

Despite the clear proof of the involvement of GST in drug resistance in nematodes, there is currently no evidence that GST expression and activity are linked with drug resistance in trematodes. For instance, RNA interference targeting the σ class of GSTs in the liver fluke *F. gigantica* causes the robust transcriptional silencing of σGST for up to 15 days of observation without any measurable changes in worm viability [70]. Because a GST knockdown in trematodes has not been shown to have any significant effect on survival in either juvenile or adult individuals, GST enzymes are not regarded as potential targets for the development of human anthelmintic drugs against trematodes.

Nevertheless, helminth GSTs are considered an important target for vaccine development [71,72,73]. In particular, a cytosolic GST from *S. haematobium* (Sh28GST) has passed phase I of clinical trials [74]. Similarly, glutathione S-transferase (SjGST) is regarded as a basis for a DNA vaccine against murine *S. japonicum* infection [73]. A SjGST DNA vaccine delivered using the nanoparticle gene delivery system exerts an antifecundity effect on female adult schistosomes and is a promising candidate for anthelmintic therapy and transmission-blocking applications [73].

### 4.3. Phase III Proteins

The inhibitors of P-glycoproteins enhance the therapeutic anthelmintic effect of drugs in vitro. In addition, it was shown that mammalian P-glycoprotein inhibitors retain their effectiveness at suppressing the helminth efflux system and even raise the sensitivity of resistant isolates. In particular, the disruption of vitellogenesis and spermatogenesis by triclabendazole (TCBZ) was revealed in a TCBZ-resistant isolate of *F. hepatica* following incubation in vitro with a P-glycoprotein inhibitor [75]. P-glycoprotein inhibitor R(+)-verapamil increases the drug susceptibility of a triclabendazole-resistant isolate of *F. hepatica* [76].

By contrast, the treatment of murine schistosomiasis with P-glycoprotein inhibitors does not improve the therapeutic effect against schistosomes. In particular, systemic treatment with ivermectin, even in the presence of the pharmacological inhibition of P-glycoprotein or cytochrome P450 3A, does not result in effective prophylaxis of *S. mansoni* infection in an experimental murine model [77].

## 5. Conclusions

In this paper, we aimed to describe structural and functional organization of the system of metabolism and transport of xenobiotics in trematodes and to compare it with available data about other species. Meanwhile, we also examined the importance of this system’s activities for the survival of helminths and potential usefulness of components of this system as targets for anthelmintic therapy.

In parasitic organisms, the need to adapt to the conditions inside the host for increasing the survival and self-reproduction is the engine behind the regression of some organs and systems and high specialization of others. The vivid examples of biochemical adaptations to parasitism via the simplification of basic metabolic systems are reductions of biosynthesis pathways of cholesterol and fatty acids and of the xenobiotic metabolism system. For example, such enzymes as flavin monooxygenases and epoxide hydrolases have been eliminated in trematodes, and the cytochrome P450 family is represented by a single gene. As for phase II, genes encoding UDP-glucuronosyl transferase and glycine-N-acyltransferase are absent, and the number of glutathione S-transferase genes is much less than that in nematodes.

The monooxygenase reactions realized by cytochromes P450 are a necessary step for physiological processes in various organisms, from protozoa to multicellular eukaryotes [10]. The proteins of this system are present in all living organisms and are involved in the metabolism of steroids, bile acids, cholesterol, unsaturated fatty acids, and phenolic metabolites; in the synthesis of prostaglandins; and in the neutralization of xenobiotics and drugs. With respect to the adult stage of the trematode life cycle, it can be assumed that adults colonizing bile ducts or blood vessels, where the components of their habitat have already passed the “barrier” of the CYP450 enzymatic system and other types of host detoxification enzymes, practically do not need a multicomponent monooxygenase system. Accordingly, two scenarios of functional and catalytic activity of CYP450 seem to be the most probable. On the one hand, this enzyme can have broad substrate specificity and participate in the metabolism of a wide range of exogenous compounds, including drugs. In this case, the biological role of CYP450 may be focused on the defense and adaptation of parasites. Alternatively, the evolutionary preservation of this enzyme may also be related to its narrow specialization within the framework of some critically important endogenous process, for example, the biotransformation of a key endogenous substrate. This hypothesis is supported by the finding that a comparison of differentially expressed genes among adult trematodes *O. felineus*, *O. viverrini*, and *C. sinensis* reveals no significant differences in the sets of genes encoding proteins of the detoxification system. This observation probably points to the conservatism of the adaptation mechanisms of these liver flukes living inside the final host [14].

It has been shown that *O. viverrini, O. felineus* and *S. haematobium* trematodes synthesize catechol estrogens and other specific metabolites of cholesterol [8,9,78], whose increased production is associated with pathogenicity to the host. Oxysterols are products of cholesterol metabolism, probably generated enzymatically with the participation of cytochrome P450 or nonenzymatically with the help of reactive oxygen species. Oxysterols are genotoxic and may be implicated in carcinogenesis [79]. Ultimately, there are reasons to believe that CYP450 and the P-glycoprotein system represent important pharmaceutical targets, both for a targeted monotherapy and for combination treatment with other officially approved anthelmintic drugs.

There are still many unanswered questions about helminth biochemistry. For instance, the endogenous functions of the biotransformation system remain unknown. In addition, how the evolutionary simplification of the xenobiotic metabolism system in trematodes affects the functionality of this system remains an important and relevant topic and requires further research. It should also be investigated how biochemical adaptation develops in an evolutionary context: whether a decrease in the number of genes is accompanied by expansion of the functions of the remaining enzyme (i.e., broad substrate specificity of the enzyme) or by high specialization of this single protein.

## Data Availability

Not applicable.
